# Colonized patients by *Candida auris*: Third and largest outbreak in Brazil and impact of biofilm formation

**DOI:** 10.3389/fcimb.2023.1033707

**Published:** 2023-01-23

**Authors:** Camylla Carvalho de Melo, Bruna Rodrigues de Sousa, Gisela Lara da Costa, Manoel Marques Evangelista Oliveira, Reginaldo Gonçalves de Lima-Neto

**Affiliations:** ^1^ Hospital da Restauração, Department of Health, State of Pernambuco, Recife, Brazil; ^2^ Post-Graduate Program in Tropical Medicine, Center for Medical Sciences, Federal University of Pernambuco (UFPE), Recife, PE, Brazil; ^3^ Post-Graduate Program in Fungal Biology, University of Pernambuco (UFPE), Recife, Brazil; ^4^ Oswaldo Cruz Institute, FIOCRUZ, Rio de Janeiro, RJ, Brazil

**Keywords:** *Candida auris*, surveillance, infection control, biofilm, outbreak

## Abstract

**Objective:**

To describe the clinical–epidemiological features of patients colonized by *Candida auris* in the largest outbreak in Brazil and to show the biofilm formation capacity of yeast strains.

**Methods:**

Clinical yeasts suspected of *C. auris* isolated from urine and surveillance samples were seeded on chromogenic media at 30°C and Sabouraud agar at 42°C. matrix-assisted laser desorption/ionization–time of flight mass spectometry was used for reliable identification. After proteomic confirmation, the genomic approach and culture on Chromagar *Candida* Plus media were carried out. Biofilm formation was investigated based on metabolic activity, and the clinical–epidemiological profile of patients was described.

**Results:**

A total of 11 C*. auris* clinical yeasts from nine patients were identified between the end of December 2021 and March 2022. Two clinical yeasts were isolates from urine and nine clinical yeasts were isolates from axillary and inguinal surveillance swabs. No case is related to previous Severe acute respiratory syndrome coronavirus 2 (SARS-CoV-2) infection. However, all the yeasts showed a high ability of biofilm formation.

**Conclusion:**

*C. auris* requires great vigilance as its high capacity to colonize and form biofilms contributes to its dissemination. The rapid and precise identification of this species is essential for the management, control, and prevention of infections.

## Introduction

*Candida auris* is an emerging opportunistic pathogen that was first reported in Japan in 2009 causing otomycosis (Satoh et al., 2009). Since then, it has been reported on all continents, with the exception of Antarctica, both as a causative agent of invasive infections and as a colonizer (Lockhart et al., 2017; Lone and Ahmad, 2019; Chow et al., 2020; Sabino et al., 2020). Associated with healthcare-related outbreaks in hospital settings, *C. auris* is reported as a public health problem due to the high mortality rates associated with bloodstream infection (Shastri et al., 2020; Briano et al., 2022).

Unlike most environmental fungi, *C. auris* is thermotolerant, growing from 37°C to 42°C. In addition, it has a remarkable ability to survive adverse environmental conditions for long periods, adapting outside the human host (Allert et al., 2022). Such facts increase the risk of hospital outbreaks as colonization and infections can originate from environmental sources, such as contaminated medical devices and the hands of health professionals (Rossato and Colombo, 2018; Ruiz-Gaitán et al., 2019; Prestel et al., 2021).

The identification of *C. auris* in the routine of a clinical laboratory can be considered problematic as conventional diagnostic procedures such as standard biochemical identification platforms/kits fail to identify such a species (Mizusawa et al., 2017; Aldejohann et al., 2022). However, if up-to-date technologies and databases are used, such as matrix-assisted laser desorption/ionization–time of flight (MALDI-TOF) mass spectrometry (MS), a sequence analysis of the D1–D2 region of 28S rDNA, or internal transcribed spacer (ITS) ribosomal DNA (rDNA), the identification of this species can be confirmed reliable (Sabino et al., 2020; Keighley et al., 2021; Aldejohann et al., 2022).

In Brazil, *C. auris* was first reported in 2020, where it was isolated as a colonizer from two hospitalized coronavirus disease 2019 (COVID-19) patients in the city of Salvador, one being from a central venous catheter tip and another from blood culture (De Almeida et al., 2021). In general, *C. auris* candidemia occurs secondary to colonization; thus, identifying this species at this stage, as well as understanding the risk factors among colonized patients, can aid in early diagnosis and prevent invasive infection (Prestel et al., 2021; Briano et al., 2022).

In this context, we present the third and biggest outbreak report of patients colonized by *C. auris* in Brazil and the first in the state of Pernambuco, northeastern Brazil. Moreover, the ability of biofilm formation from the *C. auris* clinical strains was assessed.

## Patients and methods

These reports were submitted to the Human Research Ethics Committee of the Center for Health Sciences at the Federal University of Pernambuco (UFPE, Brazil) under protocol CAAE 00480818.0.0000.5208 and received approval under protocol number 3.158.725. Informed, written consent of the patients was obtained by medical staff soon as the yeasts were identified by MALDI-TOF MS. The mapping of the clinical–epidemiological profile of the patients colonized by *C. auris* is described in **Table 1**.

**Table 1:** Mapping of the clinical–epidemiological profile of patients colonized by *Candida auris*.

Case/Isolate	Age	Gender	Underlying diseases	RT-PCR COVID-19	Place of hospitalization at time of collection	Hospital stay (days)	ICU stay (days)	Diagnostic hypothesis for hospital admission and ICU	Site of colonization	Medical devices associated with colonization	Clinical outcome
HR 01	38	M	EIL holder	Not detectable	Neurosurgery ward 504	39	–	Worsening of the general condition, hydrocephalus, PO	Urine	Indwelling bladder probe	High
HR 02 HR 02.1	70	W	DM and SAH	Not performed	Bed 28 of ICU C	42	6	Hydrocephalus, subarachnoid hemorrhage, PO, sepsis, RTI	Urine*	Indwelling bladder probe	Death
HR 03	46	M	ND	Not detectable	Bed 19 of ICU C	52	52	TBI, PO	Axillary and inguinal region	–	High
HR 04	69	M	ND	Not performed	Bed 19 of ICU C	71	71	Hemothorax, TCE, PO	Axillary and inguinal region	–	High
HR 05	40	W	DM and SAH	Not detectable	Bed 19 of ICU C	10	9	Convulsions, hemorrhagic stroke, subarachnoid hemorrhage, PO, RTI, septic shock	Axillary and inguinal region	–	Death
HR 06	41	M	ND	Not detectable	Bed 3 of ICU A	52	13	TCE, hydrocephalus, PO, hemodynamic shock, respiratory failure	Axillary and inguinal region	–	Death
HR 07	62	M	ND	Not detectable	Bed 18 of ICU B	55	48	TBI, pneumothorax, PO, LLC	Axillary and inguinal region	–	High
HR 08	24	M	ND	Not detectable	Bed 24 of ICU C	73	70	TBI, PO, RTI	Axillary and inguinal region	–	High
HR 09 HR 09.1	22	M	ND	Not performed	Bed 23 of ICU C	17	17	Gunshot wounds in the thoracoabdominal region, PO	Axillary and inguinal region*	–	Death

RT-PCR COVID-19, real-time polymerase chain reaction test for the detection of SARS-CoV-2.

M, man; W, woman.

DM, diabetes mellitus; EIL, expanding intraventricular lesion; SAH, systemic arterial hypertension; ND, not declared.

ICU, intensive care unit.

LLC, lowering level of consciousness; PO, postoperative; RTI, respiratory tract infection; TBI, traumatic brain injury.

*Same site of colonization.

### Case 1

A 38-year-old man, with expansive intraventricular lesions and with a history of tumor resection for 5 years and difficulty in swallowing, was hospitalized in November 2021 in a tertiary hospital in Recife, Pernambuco due to the worsening of his general condition after airway hypersecretivity and dyspnea, feeding by a nasoenteral tube. During hospitalization, a real-time polymerase chain reaction (RT-PCR) test for the detection of SARS-CoV-2 was performed, which was not detectable. In addition, the patient underwent the implantation of an external ventricular shunt catheter and a ventriculoperitoneal shunt catheter due to hydrocephalus. During his stay in the neurosurgery ward 504, he used an indwelling urinary catheter, and after routine examinations, he presented urinary colonization by *C. auris* (HR 01). After identifying such colonization in January 2022, the patient was asked to isolate as a contact precaution, but he had already been discharged from the hospital in December 2021 after being hospitalized for 39 days. After the fungal identification, a home visit was carried out by health staff for the screening and surveillance of colonization and a sample collection of axillary and inguinal swabs showed a negative result for *C. auris.* So far, the patient remains bedridden, with purulent diuresis and pressure ulcer in the sacral region.

### Case 2

A 70-year-old diabetic and hypertensive woman was admitted in November 2021 to a tertiary hospital in Recife, Pernambuco, due to sudden headache, vomiting, diarrhea, and fever. A CT scan of the skull revealed hydrocephalus and subarachnoid hemorrhage (Fisher IV), and urgent neurosurgery was performed. After the treatment for cerebral aneurysm embolization and cerebral vasospasm, the patient was referred to bed 28 of Intensive Care Unit C (ICU), where she was in a serious general condition, had been tracheostomized, and was using meropenem and vancomycin for the treatment of the infection of the respiratory tract. An RT-PCR test for the detection of SARS-CoV-2 was not performed. During her stay in ICU C, she used an indwelling urinary catheter and, in urine cultures collected at different times (December 2021 and January 2022), colonization by *C. auris* (HR 02 and HR 02.1) was identified. After identifying the colonization, the patient was isolated as a contact precaution and the medical staff were instructed on the need for antifungal treatment in the case of suspected invasive infection and the replacement and/or removal of the indwelling urinary catheter. It is noteworthy that this patient shared the same environment with case 1. After 42 days of hospitalization, he died due to the sepsis of a pulmonary focus, ventilator-associated pneumonia, and a lowered level of consciousness.

### Case 3

A 46-year-old man, a victim of a traumatic brain injury with signs of mental confusion, was admitted in December 2021 to a tertiary hospital in Recife, Pernambuco, and underwent a decompressive craniectomy with the drainage of an acute subdural hematoma without complications. After the surgical procedure, the patient was referred to bed 19 of ICU C, where he was hemodynamically stable, under invasive mechanical ventilation with tracheostomy, a nasoenteral tube, and peripheral venous access. Due to the presence of a confirmed case of *C. auris* in the same place of hospitalization of the patient, axillary and inguinal surveillance swabs were obtained to screen for the yeast. In January 2022, colonization by *C. auris* was evidenced in both regions (HR 03). After the identification of colonization, the patient was isolated as a contact precaution. During hospitalization, an RT-PCR test for the detection of SARS-CoV-2 was performed, which was not detectable. After 52 days of hospitalization, the patient was discharged with referral for home care and the continuity of contact precautions in case of further interventions.

### Case 4

A 69-year-old man, a victim of multiple trauma, was admitted in December 2021 to a tertiary hospital in Recife, Pernambuco, where he underwent videothoroscopies and pulmonary decortication for bilateral chest drainage secondary to hemothorax and frontotemporal contusion. After the surgical procedure, the patient was referred to bed 19 of ICU C, where he was in a serious general condition, using meropenem, invasive mechanical ventilation, a nasoenteral tube, and a indwelling urinary catheter. He also had a chest tube. As a result of the investigation of the outbreak by *C. auris* at the hospital, axillary and inguinal surveillance swabs were performed to screen. In January 2022, it was evidenced that both regions of the patient were colonized by *C. auris* (HR 04). After identification, contact precaution measures and intensified environmental and hand hygiene were taken. An RT-PCR test for detection of SARS-CoV-2 was not performed. After 71 days of hospitalization, the patient was discharged.

### Case 5

A 40-year-old diabetic and hypertensive woman was admitted in February 2022 to a tertiary hospital in Recife, Pernambuco, due to intense headache progressing to seizures and a hemorrhagic stroke due to the rupture of an arterial aneurysm in a CT scan of the skull (subarachnoid hemorrhage—Fisher IV). After the surgical procedure for the implantation of an external ventricular shunt catheter, she was referred to bed 19 of ICU C, where she had a central venous catheter in a subclavian vein, an indwelling urinary catheter, and invasive mechanical ventilation with an orotracheal tube. After 10 days of admission, there was one death due to respiratory tract infection and septic shock. During hospitalization, an RT-PCR test for the detection of SARS-CoV-2 was performed, which was not detectable. Due to admission to the ICU where *C. auris* was identified, surveillance swabs were collected to screen for the microorganism. In this patient, *C. auris* colonization can be identified on anal and axillary swabs (HR 05).

### Case 6

A 41-year-old man was admitted in February 2022 to a tertiary hospital in Recife, Pernambuco, due to trauma with hydrocephalus, where he underwent external ventricular shunt and, later, a ventriculoperitoneal shunt. After the surgical procedure, he was admitted to the neurosurgery infirmary, where several implantation and catheter removal procedures were performed. An RT-PCR test for the detection of SARS-CoV-2 was performed, which was not detectable. In the same month, he was referred to bed 3 of ICU A, and, due to the outbreak by *C. auris* at the hospital, axillary and inguinal surveillance swabs were collected, which were positive for *C. auris* (HR 06). After 52 days of hospitalization, the patient died due to hemodynamic shock, respiratory failure, and brain tumor.

### Case 7

A 62-year-old man was admitted in February 2022 to a tertiary hospital in Recife, Pernambuco, a victim of an automobile accident. After radiological examinations, severe traumatic brain injury and pneumothorax on the right were evidenced, which led the patient to undergo surgery to drain a pneumothorax. The patient was transferred to ICU B, where he remained with a closed chest tube on the right. After the stabilization of the clinical picture, he was transferred to the medical clinic sector. However, 24 h after the transfer, he was readmitted to bed 18 of ICU B with a lowered level of consciousness. During his stay in the ICU, the patient was under invasive mechanical ventilation with an orotracheal tube, central venous access in the right subclavian artery, and indwelling urinary catheter diuresis. The patient was treated with ceftriaxone, piperacillin/tazobactam, and vancomycin. Due to the *C. auris* outbreak in the hospital environment, axillary and inguinal surveillance swabs were collected and evidenced colonization by *C. auris* (HR 07). An RT-PCR test for the detection of SARS-CoV-2 was performed, which was not detectable. After 55 days of hospitalization, the patient was discharged.

### Case 8

A 24-year-old man was admitted in February 2022 to a tertiary hospital in Recife, Pernambuco, due to traumatic brain injury with diffuse axonal lesion in the thalamus on the right, found through a CT scan of the skull. After stabilization, the patient was referred to bed 24 of ICU C, where he was under invasive mechanical ventilation with tracheostomy, peripheral venous access, and a urinary device. In addition, he made use of meropenem and teicoplanin due to respiratory tract infection. An RT-PCR test for the detection of SARS-CoV-2 was performed, which was not detectable. Axillary and inguinal surveillance swabs were collected, which showed colonization by *C. auris* (HR 08). After 73 days of hospitalization, the patient was discharged.

### Case 9

A 22-year-old man was admitted in February 2022 to a tertiary hospital in Recife, Pernambuco, due to multiple gunshot wounds in the thoracoabdominal region. After the surgical procedure of exploratory laparotomy with esophagography and bilateral chest drainage, the patient was referred to bed 23 of ICU C, where he remained in serious condition under invasive mechanical ventilation with an orotracheal tube, nasogastric tube, central venous catheter, indwelling urinary catheter, and chest tubes. During this period in the ICU, axillary and inguinal swabs were collected at different times (01/03/2022 and 08/03/2022), which showed colonization by *C. auris* (HR 09 and HR 09.1). The RT-PCR test for the detection of SARS-CoV-2 was not performed. Even under treatment, the patient had a grade 2 pressure injury and an abdominal operative wound. The patient underwent a new surgical procedure, but he died after 21 days of hospitalization.

## Methods

### Sample type

After the growth of the selected *C. auris* colonies from nine patients in Sabouraud dextrose agar (SDA, Difco, Becton-Dickinson and Company, Holdrege, Nebraska, USA) at a temperature of 30°C for 24 h, it was prepared with the aid of a 10 µl sowing loop, a homogeneous suspension of each yeast in 0.9% saline solution, equivalent to the McFarland scale’s No. 1 standard. Then, a 1 µl aliquot of this suspension was seeded by the depletion technique in Petri dishes (90 mm) containing CHROMagar^®^ *Candida* (Difco, Becton-Dickinson and Company, USA) and CHROMagar *Candida* Plus™ (CHROMagar, France).

The plates were incubated at 30°C (CHROMagar^®^ *Candida*) Rio de Janeiro and 37°C (CHROMagar *Candida* Plus™) for 48 h to assess their purity and chromogenic characteristics. The interpretation of the results was based on the macroscopic observation of colonies in CHROMagar *Candida* being evaluated for the presence or absence of growth. *Candida* species with light-blue colonies and a blue halo (a color described by the manufacturer as a suggestion of *C. auris* species) on CHROMagar Candida Plus™ (CHROMagar, France) were identified as *C. auris.* The reference strain *C. auris* (derived from Centers for Diseade Control and Prevention CDC B11903) was used in both media.

### Matrix-assisted laser desorption/ionization–time of flight MS identification

The homogeneous inocula of yeast cells were grown and maintained on a yeast extract peptone dextrose (YEPD) agar medium. Incubations were standardized at 20 h, and strains were grown aerobically at 37°C. All cultures were checked for purity prior to MALDI-TOF analysis, according to Lima-Neto et al. (2014).

One single colony was directly deposited onto a spot position target plate (VITEK^®^ MS, bioMerieux, France), and two deposits were made for each isolate. The aliquots of 1 μl of 70% formic acid was added and mixed gently with the biomass deposited. When the liquid medium was almost evaporated, the preparation was overlaid with 1 μl of saturated matrix solution (75 mg/ml of α-cyano-4-hydroxycinnamic acid (CHCA) in ethanol/water/acetonitrile [1:1:1] with 0.03% trifluoroacetic acid (TFA). The mass range from 2,000 to 20,000 Da was recorded using a linear mode with a delay of 104 ns and an acceleration voltage of +20 kV. The resulting peak lists were exported by the software package SARAMIS (Kratos Analytical Ltd., Manchester, UK) where the final identifications were achieved after comparing spectra with VITEK MS Expanded 3.2 Database.

### Molecular identification

Single yeast colonies were cultured in SDA (Difco, Becton-Dickinson and Company, USA) and incubated at 30°C for 48 h, and genomic DNA were extracted from *Candida* sp. isolates in the yeast phase using the Gentra^®^ Puregene^®^ Yeast and G+ Bacteria Kit (Qiagen^®^, MD, USA), according to the manufacturer’s protocol.

Briefly, in PCR analysis, each isolate was tested with the universal fungal primers ITS1 (CGTAGGTGAACCTG CGG) and ITS4 (TCCTCCGCTTATTGATATGC) (Lindsley et al., 2001), which target the ITS1-5.8S-ITS2 region of the rDNA gene. The DNA amplification reaction was performed in a 50 µl volume containing 100 ng of yeast DNA, 10 pmol each of primers. The PCR was performed in a Veriti Thermal Cycler (model 96-Well Fast Thermal Cycler), and the annealing temperature was 58°C.

The amplicons obtained were purified with the QIAquick^®^ PCR Purification Kit (QIAGEN^®^) according to the manufacturer’s protocol. Automated sequencing was done using the Sequencing Platform at Fundação Oswaldo Cruz—PDTIS/FIOCRUZ, Brazil. The sequences were edited using the Codon Code Aligner software and compared by the basic local alignment search tool (BLAST) with sequences available from National Center for Biotechnology Information NCBI/GenBank. Phylogenetic analysis was done using the neighbor-joining algorithm (default) of Saitou and Nei (Saitou and Nei, 1987), with 1,000 replicate bootstraps, based on the alignment of the obtained ITS sequences of reference strains belonging to different clades (South Asian, East Asian, South African, South American, and Iran) deposited in GenBank.

### Evaluation of biofilm formation capacity on polystyrene surface

The method to assess the biofilm formation capacity was adapted from Pierce et al. (2008) and Ruiz et al. (2013). Yeasts were grown in yeast extract peptone dextrose (YPD) at 37°C overnight and then suspended in RPMI Medium 1640 buffered with HEPES (Gibco^®^) and adjusted to a concentration at 10^6^ cells/ml. Subsequently, 100 µl were added to the wells of polystyrene plates containing 96 wells and incubated in an incubator with shaking at 75 rpm at 37°C for 2 h. Then, the wells were washed three times with phosphate-buffered saline (PBS) to remove non-adherent cells and incubated for 24 h at 37°C for further evaluation. The isolates of *C. albicans* American Type Culture Collection ATCC 90028 and *C. auris* CDC B11903 were used as a control.

The quantification of biofilms was performed with the salt reduction assay of 1-(4,5-dimethylthiazol-2-yl)-3,5-diphenylformazan (MTT; Sigma Chemical, St. Louis, MO, USA), where 20 µl, in the proportion of 5 µl–1 ml of a PBS buffer, sterilized by membrane filtration (Millipore, 0.22 µm pores) was added to each well of the microtiter plate, including controls. The plates were incubated in the dark at 37°C for 18 h. Subsequently, the dye was aspirated, and then, 200 µl of isopropanol was added (Freimoser et al., 1999; Berridge et al., 2005). The plates were left to rest for 15 min, and 100 µl of the contents of each well were transferred to a new microtiter plate in order to read them in a microplate reader with a wavelength of 570 nm. The biofilm production in *Candida* species was categorized as high (Optical Density OD_570_≥0.1), low to moderate (0.025<OD_570_<0.1), or absent (OD_570_ ≤ 0.025) (Pannanusorn et al., 2013).

For statistical analysis, the software GraphPad Prism 8.0.1 (GraphPad Software, Inc., La Jolla, CA, USA) was used. The data obtained were verified for their normal distribution and were expressed as the mean ± standard error of three replications. Data were compared using two-way analysis of variance with multiple comparisons by Tukey’s *post hoc* test. The *p*-value <0.05 was considered significant.

## Results

### Strain identifications

**Figure 1:**
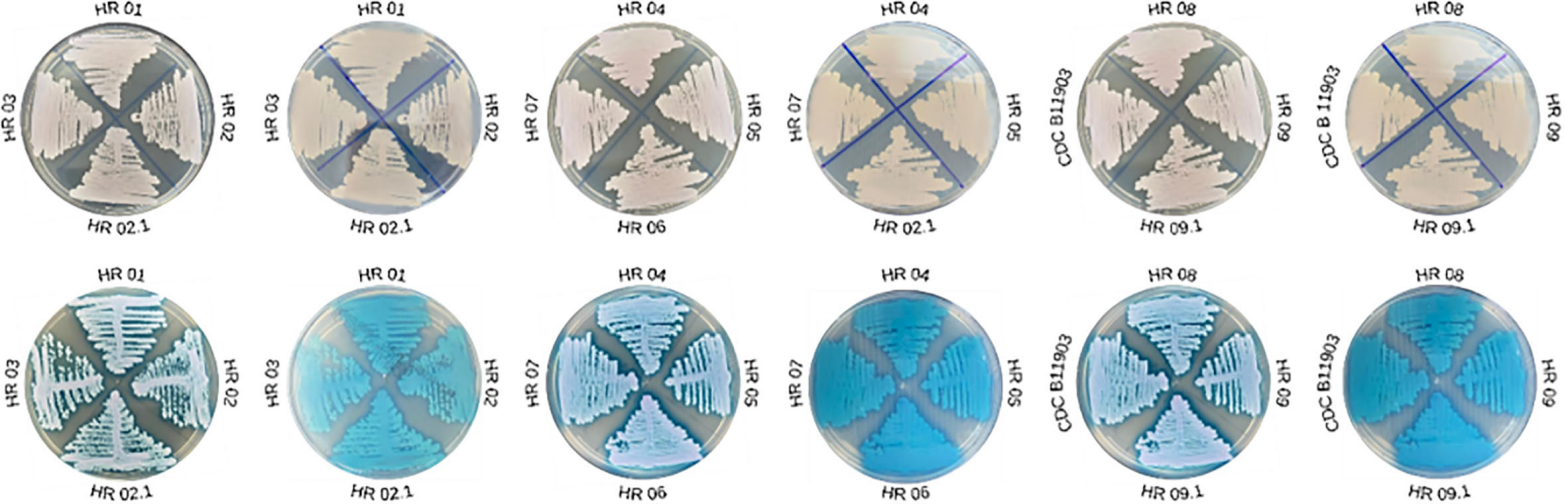
Growth of *Candida auris* clinical isolates on conventional CHROMagar^®^ *Candida* (Difco, Becton-Dickinson and Company, USA) versus CHROMagar *Candida* Plus™ (CHROMagar, France).

The isolates obtained in our study yielded colonies that stained in the chromogenic medium CHROMagar^®^ *Candida* (Difco, Becton-Dickinson and Company, USA) and showed color white and pink tone. However, in all samples, there was one color only, demonstrating that there were no mixed colonies (**Figure 1**). Likewise, in CHROMagar *Candida* Plus™ (CHROMagar, France), the species identification was possible according to the manufacturer’s guidelines, as the 11 C*. auris* clinical isolates showed the color standard from this species light blue with blue halo (**Figure 1**).

**Figure 2:**
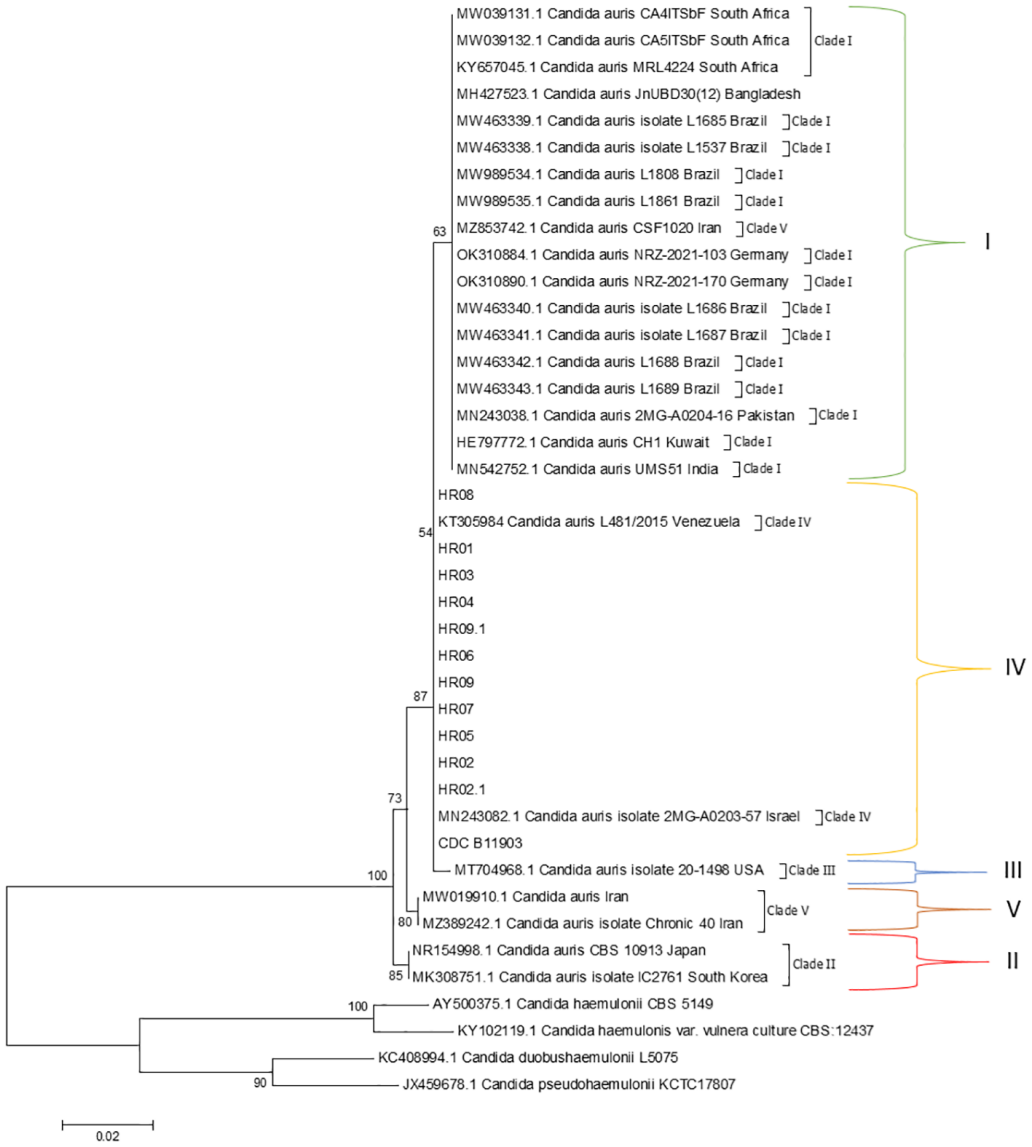
Evolutionary relationships of taxa. Phylogenetic tree generated by neighbor-joining, maximum likelihood, and maximum parsimony analysis using partial nucleotide sequences of ITS. Bootstrap values (1,000 replicates) were added to the respective branches. All species are indicated at its respective position at the phylogenetic tree. Evolutionary relationships involved five taxa and the analysis of 19 nucleotide sequences by six *C. auris* strains of previous studies used as a reference to each clade reported in this species, in which sequences were obtained from GenBank. There were a total of 250 positions in the final dataset. Evolutionary analyses were performed in MEGA6 (Tamura et al., 2013).

MALDI-TOF MS identified the 11 clinical yeasts reliably as *C. auris*, all with a percentage of 99.9%. In PCR amplification with the ITS region and sequenced, the 11 isolates were characterized as *C. auris* when compared, available from NCBI/GenBank (*C. auris* KT3059841) (**Figure 2**). The sequences of the isolates have been deposited in GenBank under the following accession numbers: HR 01 (ON256389), HR 02 (ON256397), HR 02.1 (ON256398), HR 03 (ON256390), HR 04 (ON256391), HR 05 (ON256396), HR 06 (ON256393), HR 07 (ON256395), HR 08 (ON256388) HR 09 (ON256394), and HR 09.1 (ON256392).

### Biofilm formation capacity on polystyrene surface

**Figure 3:**
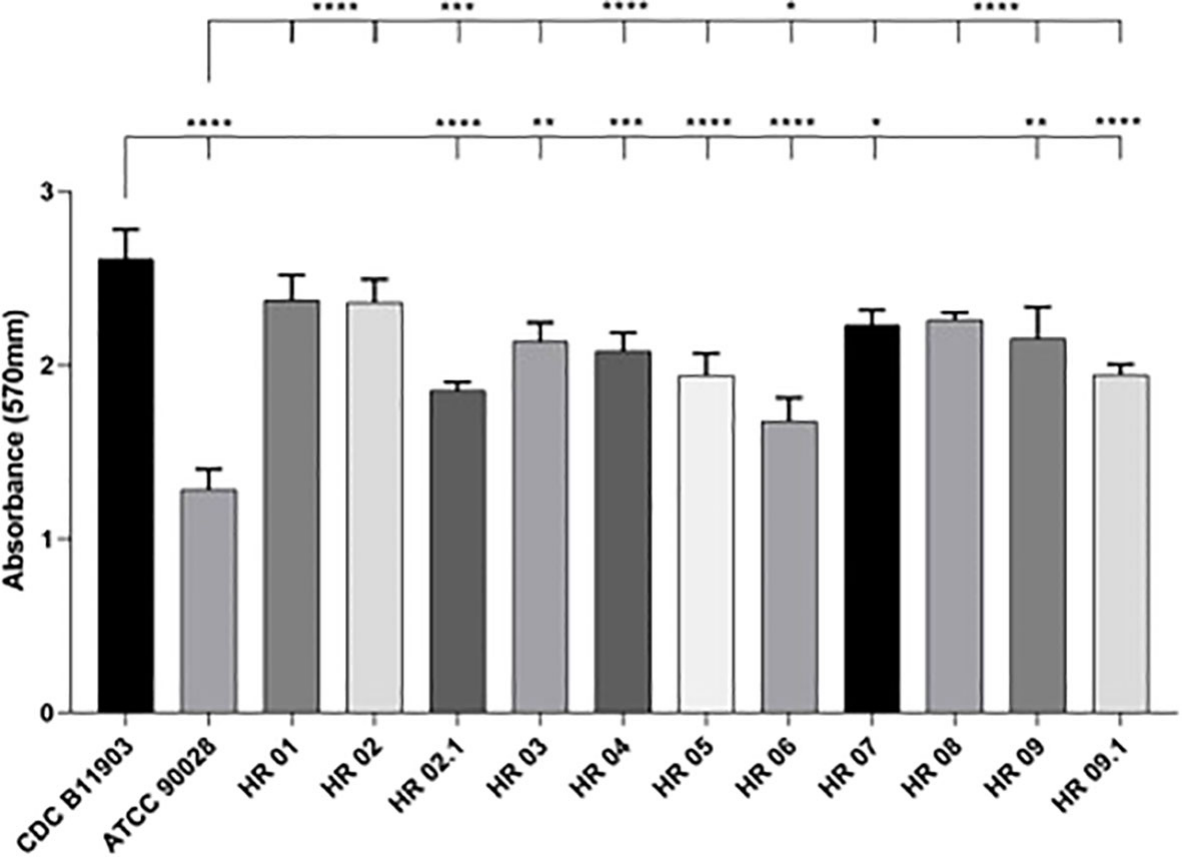
Biofilm formation capacity on the polystyrene surface of clinical isolates of *C. auris* after 24 h. One-way analysis of variance with multiple comparisons by Tukey’s *post hoc* test was used to determine statistical significance. The values of p < 0.05 were considered significant: *<0.05; **<0.01; ***<0.001; ****<0.0001.

The biofilm quantification based on metabolic activity revealed that all *C. auris* colonizing isolates were able to form active biofilms within 24 h, which were categorized as strong producers. The values of the formed biofilms are summarized in **Table 2** and **Figure 3**. In comparison with *C. auris* CDC B11903, no strain in the study showed a higher biofilm production after 24 h of incubation. However, HR 01, HR 02, and HR 08 strains showed high OD values and no statistical difference in relation to this *C auris* reference strain. It can be seen as a different result associated with biofilm formation by *C. albicans* ATCC 90028, where all the clinical isolates presented a higher biofilm formation, with highlight on the *C. auris* HR 01, which was able to form a biofilm 1.8× larger (p>0.0001). The isolate HR 06 produced the smaller biofilm and statistically differed with *C. auris* CDC B11903 and *C. albicans* ATCC 90028 with the *p-*values of <0.0001 and <0.05, respectively.

**Table 2:** Biofilm formation capacity on the polystyrene surface of clinical isolates of *C. auris* in 24 h.

Clinical isolates	Biofilm formation*	Pattern^#^
HR 01	2.376	High
HR 02	2.361	High
HR 02.1	1.854	High
HR 03	2.137	High
HR 04	2.083	High
HR 05	1.944	High
HR 06	1.679	High
HR 07	2.233	High
HR 08	2.261	High
HR 09	2.156	High
HR 09.1	1.947	High
CDC B11903	2.613	High
ATCC 90028	1.283	High

*Data are expressed as OD570 and represent the mean absorbance of 1-(4,5-dimethylthiazol-2-yl)-3,5-diphenylformazan during triplicate biofilm production after 24 h.

^#^Biofilm formation pattern categorized as high (OD_570_≥0.1), low to moderate (0.025<OD_570_<0.1), or absent (OD_570_ ≤ 0.025) (Pannanusorn et al., 2013).

## Discussion

In December 2022, 2 years after the first *C. auris* outbreak inpatients hospitalized in Salvador (Bahia State, Brazil), the growth of yeasts identified as *C. auris* took place in a public hospital in the city of Recife, state of Pernambuco, Brazil. It is the largest public hospital in the Northeast region with 704 beds and the reference in trauma, general surgery, oral and maxillofacial surgery, neurosurgery, neurology, internal medicine, and orthopedics.

*C. auris* infections have been emerging across the globe, often with associated high morbidity and mortality. Then, the availability of methodologies fast and with accurate species-level identification are mandatory for these cases, enabling the implementation of appropriate prevention and control measures quickly. In our study, we report the applied polyphasic taxonomy in a network that made possible a fast detection of the cases of colonization by *C. auris*. As can be seen in the dendrogram, basic local alignment search tool analysis suggested *C. auris* as the etiologic agent in all cases, and phylogeny confirmed species identification in the same branch of *C. auris* L481/2015 from Venezuela classified in Clade IV.

The ability to persistently colonize hospital environments, equipment, and patient body sites by *C. auris* is particularly well known, indicating a potential involvement of this species in the formation of biofilms (Kean et al., 2018; Singh et al., 2019; Horton and Nett, 2020). Commonly, these communities are problematic and contribute to the pathogenicity of the species as they are difficult to eliminate and may result in nosocomial infections (Sherry et al., 2017; Horton and Nett, 2020).

The ability to produce biofilm is a key driver of *C. albicans* virulence. Sherry et al. (2017) argued that *C. auris* is unable to form biofilms equivalent to *C. albicans*. Nevertheless, in our study, all *C. auris* clinical strains produced more biofilm than the *C. albicans* reference strain (ATCC 90028). Interestingly, the *C. auris* reference strain (CDC B11903) produced more biofilm than all the colonizing strains evaluated.

Singh et al. (2019) compared the biofilm‐forming capacity of *C. auris* isolated by colonizing sites and candidemia. They concluded that colonizing isolates were predominantly aggregative, with a higher biofilm‐forming capacity than non‐aggregative ones. *C. auris* displayed phenotypic variation, with non‐aggregative isolates being more common among candidemia cases. This may partially explain the organism’s remarkable ability to persistently colonize the hospital environment, equipment, and patient body sites, indicating a potential involvement of biofilm formation according to our results. Furthermore, the authors showed that the majority of the *C. auris* colonizing isolates exhibit biofilm biomass similar to *C. albicans*.

An interesting and well-performed study published by Horton and Nett (2020) analyzed *C. auris* growth in a synthetic sweat medium planned to mimic axillary skin circumstances. The researchers found that *C. auris* showed a high capacity for biofilm formation, well beyond that observed for *C. albicans*. We assent with authors, who point that the capacity to thrive in skin niche conditions support the explanation about the tendency of *C. auris* to colonize skin and rapidly spread among patients.

We believe that this high formation of communities adherent to surfaces contributed strongly to the dissemination of the strains in the hospital environment due to the fact that this characteristic contributes to ecological adaptation and survival when competition for resources is high.

According to Zhu et al. (2020), little is known about the environmental survival and spread of *C. auris* strains. We agree with these authors, who argue that colonization on both biotic (patients) and abiotic (health assistance devices) surfaces and human mobility appear to be the major factors for the spread of this pathogen across the globe. Having said that, we believe that the determination of the genotypes of all clinical and environment isolates may help to investigate the source of cases reported here.

Hitherto, new cases continue to be detected by surveillance samples, and on 07/11/2022, the public health authorities of the state of Pernambuco released the notification of 47 cases and four environmental yeasts counting 51 C*. auris* isolates, featuring the largest outbreak in Brazil. It is noteworthy that *C. auris* has been isolated from the bed rails of the same hospital environment as the nine patients described here. We hypothesized that the high capacity to form biofilms is associated with the maintenance of yeast on surfaces and measures related to hygiene must be taken.

## Data availability statement

The datasets presented in this study can be found in online repositories. The names of the repository/repositories and accession number(s) can be found below: https://www.ncbi.nlm.nih.gov/genbank/, ON256389; https://www.ncbi.nlm.nih.gov/genbank/, ON256397; https://www.ncbi.nlm.nih.gov/genbank/, ON256398; https://www.ncbi.nlm.nih.gov/genbank/, ON256390; https://www.ncbi.nlm.nih.gov/genbank/, ON256391; https://www.ncbi.nlm.nih.gov/genbank/, ON256396; https://www.ncbi.nlm.nih.gov/genbank/, ON256393; https://www.ncbi.nlm.nih.gov/genbank/, ON256395; https://www.ncbi.nlm.nih.gov/genbank/, ON256388; https://www.ncbi.nlm.nih.gov/genbank/, ON256394; https://www.ncbi.nlm.nih.gov/genbank/, ON256392.

## Ethics statement

The studies involving human participants were reviewed and approved by Human Research Ethics Committee of the Center for Health Sciences at the Federal University of Pernambuco. The patients/participants provided their written informed consent to participate in this study. Written informed consent was obtained from the individual(s) for the publication of any potentially identifiable images or data included in this article.

## Author contributions

CM: data collection; acquisition, analysis, and interpretation of data; and drafting of the work. BS: acquisition, analysis, and interpretation of data and drafting of the work. GC: acquisition, analysis, and interpretation of data and drafting of the work. MO: study design; acquisition, analysis, and interpretation of data; and final approval of the version to be published. RL: study design; acquisition, analysis, and interpretation of data; final approval of the version to be published; and funding acquisition. All authors contributed to the article and approved the submitted version.
